# How to increase public participation in advance care planning: findings from a World Café to elicit community group perspectives

**DOI:** 10.1186/s12889-019-7034-4

**Published:** 2019-06-03

**Authors:** Patricia D. Biondo, Seema King, Barinder Minhas, Konrad Fassbender, Jessica E. Simon, on behalf of the Advance Care Planning Collaborative Research and Innovation Opportunities Program (ACP CRIO)

**Affiliations:** 1 Advance Care Planning Collaborative Research and Innovation Opportunities Program (ACP CRIO), University of Calgary, Calgary, Alberta Canada; 2 Covenant Health Palliative Institute, Edmonton, Alberta Canada; 3 Department of Oncology, University of Alberta, Edmonton, Alberta Canada; 4 Departments of Oncology, Medicine, and Community Health Sciences, University of Calgary, Calgary, Alberta Canada

**Keywords:** Advance care planning, World Café, Community engagement, Community groups, Public

## Abstract

**Background:**

In 2014, Alberta, Canada broke new ground in having the first provincial healthcare policy and procedure for advance care planning (ACP), the process of communicating and documenting a person’s future healthcare preferences. However, to date public participation and awareness of ACP remains limited. The aim of this initiative was to elicit community group perspectives on how to help people learn about and participate in ACP.

**Methods:**

Targeted invitations were sent to over 300 community groups in Alberta (e.g. health/disease, seniors/retirement, social/service, legal, faith-based, funeral planning, financial, and others). Sixty-seven participants from 47 community groups attended a “World Café”. Participants moved between tables at fixed time intervals, and in small groups discussed three separate ACP-related questions. Written comments were captured by participants and facilitators. Each comment was coded according to Michie et al.’s Theoretical Domains Framework, and mapped to the Capability, Opportunity and Motivation behavior change system (COM-B) in order to identify candidate intervention strategies.

**Results:**

Of 800 written comments, 76% mapped to the Opportunity: Physical COM-B component of behavior, reflecting a need for access to ACP resources. The most common intervention functions identified pertained to Education, Environmental Restructuring, Training, and Enablement. We synthesized the intervention functions and qualitative comments into eight recommendations for engaging people in ACP. These pertain to access to informational resources, group education and facilitation, health system processes, use of stories, marketing, integration into life events, inclusion of business partners, and harmonization of terminology.

**Conclusions:**

There was broad support for the role of community groups in promoting ACP. Eight recommendations for engaging the public in ACP were generated and have been shared with stakeholders.

## Background

Advance care planning (ACP) is a process that supports adults at any age or stage of health in understanding and sharing their personal values, life goals, and preferences regarding future medical care. The goal of ACP is to help ensure that people receive medical care that is consistent with their values, goals and preferences during serious and chronic illness [1]. As such, ACP is a key component of achieving person- and family-centred healthcare. ACP has been shown to improve quality of life and end of life care for individuals, to reduce decision-making burden, suffering and bereavement distress of family members, and to improve efficiency and cost shifting within the healthcare system as a whole [2–4]. Alberta, Canada is a Western Canadian province with a population of 4 million residents, and our provincial healthcare system broke new ground in having the first province-wide policy and procedure for ACP [5], initiated in 2014. This includes a medical order framework, called Goals of Care Designations (GCD), for communicating a person’s goals and guiding the associated approach to his/her medical care. GCD replaced less nuanced medical orders such as “Do Not Resuscitate.” However, despite the provincial rollout of the policy and the healthcare system’s efforts to socialize it, to date public participation and awareness of ACP and GCD remains limited (only 27% of Albertans have heard of ACP) [6]. To catalyze new community engagement in ACP activities and awareness, the Advance Care Planning Collaborative Research and Innovation Opportunities research program (ACP CRIO) hosted two World Café events to learn from community members “How can we help Albertans learn about and participate in Advance Care Planning?”

## Methods

### The World Café

The World Café is a method for hosting large group dialogue around “questions that matter” [7]. Set in a comfortable and welcoming ‘café’ environment, small groups of 4–5 participants ponder and discuss questions of interest. At regular time intervals, participants move between groups and tables, to build on and link previously voiced ideas in evolving rounds of dialogue. At the end of the café, ideas and insights are ‘harvested’ and explored with all participants through a large group conversation, in order to capture the collective wisdom of the group.

### Recruitment

Targeted invitations were sent by e-mail to over 300 community groups/organizations in Alberta. A purposeful sampling procedure, using internet searches to identify community groups, was used to target a broad range of community groups for whom ACP might be relevant e.g. health/disease, seniors/retirement, social/service, legal, cultural, recreational, faith-based, funeral planning, financial, and other groups. Participants were invited by email to attend the World Café activity in either Calgary or Edmonton, Alberta in March 2016.

### Conduct

Each café was held on a weekday afternoon at a public, community-based meeting venue, and was facilitated by a number of ACP CRIO research team members and their health services colleagues. The World Cafés began with a welcome lunch, followed by a keynote presentation on ACP to provide an introduction and ensure a common understanding of ACP among participants. A brief ‘Conversation Cookies’ [8] exercise was then conducted as a warm-up to the topic, after which participants moved into the World Café activity, where they explored three ACP-related questions (Table 1). Questions were developed through consensus by the research team/café facilitators. In small groups (~ 4–5 people per table), participants spent approximately 20 min discussing each question, and were encouraged to record or doodle comments/questions/concerns anonymously on colored placemats at each table. Café facilitators acted as table hosts to help keep groups on topic, to encourage participation from all participants (via discussion or written comments), and in some cases to act as scribes. When cued by the timekeeper, participants moved between tables and groups until all participants had explored all three questions. Café facilitators remained at their tables to summarize the previous dialogue to the incoming group and foster continuity of the conversation. A coffee break was held at the end of the World Café activity, during which café facilitators collected the colored placemats for analysis and ‘harvesting’ of ideas and insights. Frequently expressed ideas were captured for presentation back to the large group. These were explored in a large group conversation after the coffee break in order to gather collective input on the emerging themes. Written notes of this large group discussion were recorded by café facilitators.

**Table 1:** World Café Questions

	Calgary	Edmonton
Q1^a^	“Before today, how have you or your community group learned about Advance Care Planning and Goals of Care Designations?”	“What is needed for Advance Care Planning to become a ‘routine’ activity for adults?”
Q2	“What concerns might you have about promoting or encouraging community members to participate in Advance Care Planning?”	
Q3	“What could your community group do, and what would you need help with, to share Advance Care Planning information with your community?”	

^a^Q1 was modified for the Edmonton World Café as saturation was quickly achieved in Calgary

### Ethics

This activity was conceived as a community forum with the primary purpose of developing a community engagement strategy for ACP in Alberta, including recommendations for local stakeholders for engaging Albertans in ACP. Guided by Canada’s *Tri-Council Policy Statement: Ethical Conduct of Research Involving Humans, 2nd edition (TCPS 2)* [9], and the use of the Alberta Research Ethics Community Consensus Initiative (ARECCI) Ethics Screening Tool [10], review by the University of Calgary Conjoint Health Research Ethics Board was deemed unnecessary. All written comments and notes were captured anonymously (i.e. no names or otherwise identifying information were linked to any of the written notes). Participants were informed at the start of the meeting that their written notes would be analyzed and used to create a final report from the activity. Informed consent was not obtained from participants verbally or in writing, but was implied from their continued participation in the meeting. Participants were also free to abstain from leaving written comments.

### Data analysis

#### Thematic analysis by World Café question

The written comments aligning with each of the World Café questions were independently coded and themed into categories by two of the co-authors (SK, BM), in order to identify common themes in responses to each question. Categories were further reviewed and discussed amongst the research group (SK, BM, PB, JS) until consensus was reached. Just under 800 written comments in total were analyzed. Twenty-eight comments were ambiguous and could not be themed into any category.

#### Quantitative analysis using the theoretical domains framework and behavior change wheel

The written comments were also analyzed according to Michie et al.’s Theoretical Domains Framework (TDF) [11, 12] and Behavior Change Wheel (BCW) [13] as a theoretical underpinning to the development of intervention strategies for increasing ACP participation. The TDF is an integrative framework of behavior change theories and constructs, grouped into 14 theoretical domains to explain behavior change (e.g. knowledge, skills, social influences, emotion, etc.). The BCW is a synthesis of 19 frameworks of behavior change found in the research literature [12]. The BCW has at its core a model that recognizes behavior as an interacting system involving three components: Capability, Opportunity and Motivation (COM-B) [13]. The outer layers of the wheel include nine intervention functions and seven policy categories that may be chosen to support behavior change, once the sources of behavior are better understood. Thus, the COM-B behavior system and BCW provides a model for designing interventions aimed at behavior change. Each domain of the TDF has been mapped to a COM-B component by behavior change experts [11], such that the TDF can be used to expand on COM-B components to develop a more detailed understanding of the behavior to be changed [14].

Each written comment was independently mapped to one of the 14 TDF domains by two co-authors (SK, BM), who then met and resolved differences through discussion. Written comments were then mapped to their appropriate COM-B component based on their TDF domain [11]. To guide the development of intervention strategies for increasing public participation in ACP, two co-authors (SK, BM) independently identified one or more of the nine intervention functions from the BCW [12] for each written comment, appropriate to the comment’s COM-B and TDF domain; again, discrepancies were resolved through team discussion (PB, JS).

In order to translate the candidate intervention functions into practical intervention strategies, we drew on the themes generated through the qualitative data analysis to generate specific recommendations (intervention strategies) for the most frequently identified behavioral issues and intervention functions.

## Results

### Participants

**Fig. 1:**
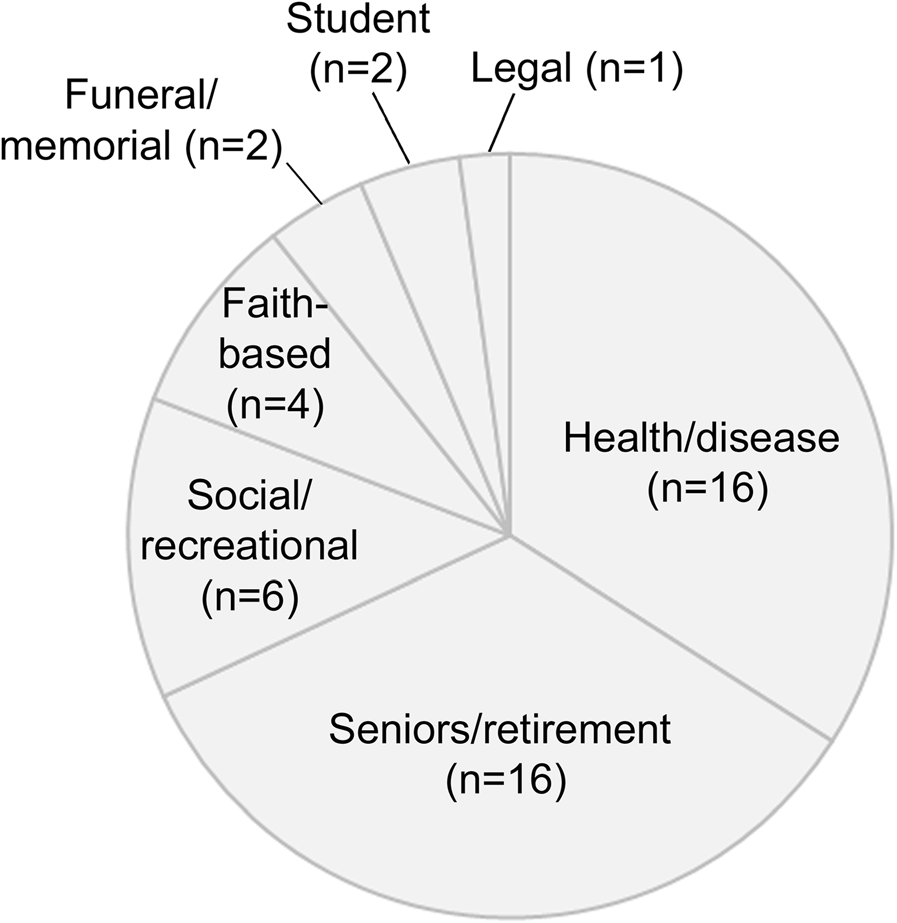
Categories of community groups attending the World Café

A total of 67 participants (46 in Calgary and 21 in Edmonton), from 47 community organizations (Fig. 1), attended the cafés. As this activity was conceived as a community forum and not a research project, specific demographic data such as age, gender, education, or other typical sociodemographic variables were not collected from participants. However, we are aware that the majority of participants were female. All participants were English-speaking.

### Thematic analysis by World Café question

#### Prior learnings about ACP

Thematic analysis of Question 1 (Calgary) indicated that prior to attending the World Café activity, participants had learned about ACP through personal experiences such as planning for their own care or care of a loved one, through presentations to their community groups, through their employment (e.g. as an “intake worker” for a seniors resource society), or during funeral and/or estate planning. Café facilitators identified that saturation was quickly reached with this question during the first café (Calgary), and so the question was replaced for the Edmonton café (Table 1).

#### Concerns about promoting ACP via community groups

Thematic analysis of Question 2 identified a variety of concerns with regard to encouraging participation in ACP, including concerns related to process, public understanding of ACP, legal and emotional issues, ACP necessity, access to the healthcare system and healthcare providers, documentation in Alberta, and the various roles involved (Table 2).

**Table 2:** Participant concerns about encouraging participation in ACP

Theme	Supporting comments
Process concerns	
• Difficulty maintaining up-to-date information	“if you have complex health problem- you don’t know how your experience, health can change”
	“emergency contact person is no longer current”
• Documents/agents inaccessible	“emergency contacts not available at time of need”
	“no one brings Green Sleeve in”
• Patient signature not required on GCD order	“GCD not signed by patient- would want to sign GCD”
• Complicated documentation	“too many forms to fill out - not friendly”
	“going through Green Sleeve is complicated”
• Authority concerns – doctors vs. agents	“want a GOC but don’t want my PD agent to lose decision making authority to a doctor of GOC document”
• Lack of support/facilitation	“need hand holding, guidance to fill in documents”
	“need help from clinician- these are the things you have to think of”
• Family/agent conflicts	“disagreement of family members – tension”
• Role confusion	“Where do we go to have these conversations?”
	“Specialists e.g. cancer care are not the medical persons to be expected to have conversations”
• Capacity issues	“If adult child with mental illness goes in and out of “capacity” how does personal directive change?”
Lack of public understanding of ACP	
• Lack of knowledge/ resources	“not enough knowledge and tools – wider availability”
	“lack of knowledge of Green Sleeve”
• Terminology is complex and/or always changing	“Language change- DNR to GCD”
	“need for plain language”
• Health literacy	“Health literacy needs to be addressed! How is a personal directive different from a power of attorney?”
Legal concerns	
• Document legality	“Confusion between GOC/PD – which overrules?”
• Jurisdiction	“Laws may be different in other countries/provinces when health failure happens - how to bring/uphold person’s ACP done in Alberta?”
• Legal costs	“People think they need to have a lawyer to get a personal directive – legal fees”
Emotional concerns	
• Uncomfortable topic	“dying - nobody wants to talk about this”
• Don’t want to destroy hope	“caregivers don’t want to broach the topic with newly diagnosed family member”
Lack of need	“people don’t think they need it”
	“not going to happen to us”
Lack of access	“Opportunity to discuss not available for everyone”
	“reaching isolated older adults”
Healthcare providers’ time constraints	“Doctors [have] no time to discuss with people. How does this happen within a 1/2 h allotment during a doctor visit?”

*DNR* do not resuscitate, *GCD* goals of care designation(s), *GOC* goals of care, *PD* personal directive

#### Suggestions for normalizing ACP in Alberta

Thematic analysis of Question 1 (Edmonton) revealed a wide variety of suggestions from participants for how to encourage ACP as a routine activity for all Albertans (Table 3). These ranged from increasing education and marketing activities, to integrating ACP into life events (e.g. with driver’s license, childbirth, retirement), to incentivising or even mandating ACP. A diverse group of community organizations were identified as being relevant to ACP promotion. These included legal organizations, disease/illness groups, funeral homes, homeless shelters, caregiver associations, seniors’ centres, primary care networks, unions, financial organizations, and faith-based groups.

**Table 3:** Suggestions for ‘normalizing’ ACP among Albertans

Theme	Supporting comments
Education	
• Educate the general public	“education of public where to access materials and how to use them”
• Educate youth/young adults	“this should be discussed in high school … to become a natural thing to look at”
• Educate professionals (e.g. healthcare, legal, financial professionals)	“Train all helping professionals in their university training about Advanced Care Planning but also trained in how to have the conversations - > skill needed”
• Educate community organizations	“holding sessions through community groups, churches, etc. to educate and having someone there to answer questions”
• Educate through conferences, health fairs	“Pushing concept at conferences- e.g. retired teachers”
	“health fairs- hand out pamphlets”
• Educate through employers	“workshops offered by companies for employees”
Integrate into life events	“Revisit PD at marriage, kids, divorce, disease, losing a family member”
• Driver’s license, marriage, birth of child, divorce, new medical diagnosis, retirement, will/estate planning, funeral planning/death of loved one	
	“Part of annual visit with GP”
	“driver’s license or health care card - have you signed a personal directive - tick box”
Introduce ACP earlier in life	“start this conversation with people when they are young”
Standardize terminology across the country	“GCD & DNR, common language in the country”
Mandate ACP	“mandate primary care networks to have conversations”
	“long term care/assisted living facilities could insist on having this for all residents”
Change focus toward quality of life, not just end of life	“frame as planning for life not planning for death”
Provide incentives (for public and professionals)	“extra lines (directives) on financial and investment and mortgage papers and life insurance policies 'personal directive discount”
• Financial, emotional	
	“change GP billing codes”
	“Educate what happens when you don’t have a PD - give the negatives of the story”
Advertise ACP	“need to advertise, let people know to normalize the activity”
• Web, social media, television, print media, annual events (e.g. National ACP Day, fun run), public awareness campaign	“put on facebook, social media for younger generation”
	“Terry Fox run, incorporate death and dying”
Make ACP accessible to different cultures	“cultural community and group education”
	“cultural training/sensitivity to ACP”
	“language translation is needed for other groups”
Share stories	“peer-to-peer stories help having experienced it already”

*DNR* do not resuscitate, *GCD* goals of care designation(s), *GP* general practitioner, *PD* personal directive

#### What community organizations can do to share ACP with Albertans

Thematic analysis of Question 3 suggested there was broad support for the role of community groups in promoting ACP in Alberta. From the nearly 800 comments themed and categorized, only two comments countered this role. One comment suggested that ACP would not be well received by the participant’s organization, and the other comment questioned the capacity of voluntary community organizations to “light a fire” around this topic.

Participants were keen to suggest ways in which their community organizations could help share ACP with their members e.g. hosting education sessions; upskilling their own members; disseminating resources such as Green Sleeves (the green plastic folder used in Alberta, Canada to house a person’s advance care planning documents) and personal directives (sometimes referred to as advance directives in other jurisdictions); sharing ACP content through websites, e-mails, social media, and newsletters; movie/video showings. A number of strategies were suggested to better enable community organizations to share ACP with their members (Table 4).

**Table 4:** Suggestions for enabling community organizations to share ACP with Albertans

Theme	Supporting comments
Provide educational opportunities for community organizations	“we could use help with having a medical practitioner have a workshop for persons needing to fill out their medical wishes more in depth”
• Seminars, guest speakers, hands-on workshops, lunch-and-learns, one-to-one education	
	“would like lunch and learns, bring in a speaker”
	“provide educators to address issues around power of attorney, personal directives and goals of care”
Provide ACP training for community organization members e.g. train-the-trainer programs	“could train people in our group to speak”
	“train-the-trainer would increase [our organization’s] comfort level”
Provide resources	
• Print resources (e.g. Green Sleeves, Personal Directives, bookmarks, posters, toolkits, pamphlets, conversation tip sheets)	“having green sleeves available for groups to order”
	“personal directive kits”
	“having a cheat sheet on things to discuss (e.g. funeral plans, mental illness, incapacity, personal directive)”
• Media resources (e.g. websites/website content, magazine articles, newsletter pieces, videos/movies/TED talks, presentation slide decks)	“series of articles 150–200 words”
	“copies of ads for community newsletters/websites”
	“powerpoints/usb keys with talks for groups to use”
	“having resources (videos)”
	“have ads on facebook for people to share”
• Personnel (e.g. speakers, dedicated ACP facilitators, telephone consultants similar to HealthLink consultants)	“provide a list of speakers to community groups”
	“have an assigned/educated facilitator/resource person for communities/facilities to access to have the advance care planning conversation”
	“have a phone number like health link where people can actually be reached to answer questions”

### Quantitative analysis using the COM-B behavior change model and behavior change wheel

**Fig. 2:**
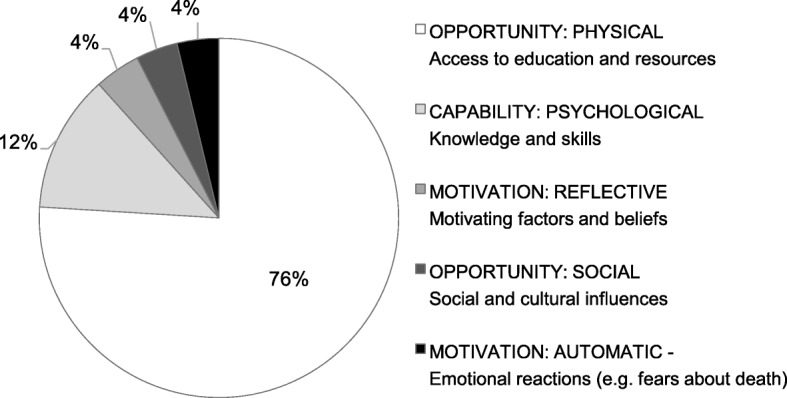
Frequency of comments mapped to the COM-B components of behavior

On mapping the written comments to the COM-B behavior change model, we found that the majority of comments (76%) mapped to the Physical Opportunity behavior component (Fig. 2).

The four most common intervention functions identified through the mapping exercise pertained to education (37%), environmental restructuring (26%), enablement (14%), and persuasion (10%). Fewer comments mapped to interventions relating to training (6%), modelling (3%), coercion (1%), incentivisation (1%) and restriction (1%).

### Recommendations for engaging Albertans in ACP

A synthesis of the most frequently identified intervention functions and the themes identified through the qualitative comments yielded eight recommendations (intervention strategies) for engaging Albertans in ACP (Table 5).

**Table 5:** Synthesized recommendations for engaging Albertans in ACP

Recommendation/Intervention Strategy	Intervention function
Make ACP resources easily accessible to community groups.	Environmental restructuring, Enablement
Make Green Sleeves and personal directive kits freely available and easily accessible to community groups. Prepare ACP content for use in newsletters, magazines, on websites, and with social media.	
Provide education and facilitation opportunities for community groups and professionals.	Education, Training, Enablement
(a) Education: Incorporate ACP into education curricula for secondary school students, into the professional education of relevant post-secondary students, into continuing professional education (e.g. medical, nursing, social work, legal, financial planning, insurance, funeral planning, etc.), and into education opportunities for relevant voluntary sectors.	
(b) Facilitation: Provide group facilitated sessions, where members come together to learn about and initiate Advance Care Planning. Create a ‘speakers bureau’ of professionals and community volunteers willing to facilitate such sessions.	
Simplify healthcare system processes and increase support for conversations	Environmental restructuring, Training, Enablement
Simplify language of resources and include explanations of how personal directives, wills, power of attorney, goals of care designations and ACP conversations relate together. Increase physician and primary care team capacity to address ACP and have the time and skills to have ACP conversations (e.g. including facilitated groups or individual coaching on how to complete documentation that reflects the person’s preferences or values).	
Use stories/make use of personal experiences	Persuasion, Modelling
Resources aimed at the public should include stories and invite reflection on prior personal experiences.	
Increase marketing of ACP to the public	Education, Persuasion, Modeling
Advertise ACP through websites, social media, television, print media, and annual events.	
Capitalize on opportunities to integrate ACP into major life events	Environmental restructuring, Incentivisation, Persuasion
Include information on ACP and Goals of Care Designations with driver’s license, with marriage license, in childbirth package, upon retirement, during will/estate planning, and other life events.	
Include business partners in ACP	Environmental restructuring, Education, Modelling
Make ACP resources accessible for use by financial planners, insurance brokers, lawyers, funeral homes and other relevant businesses for inclusion in their periodicals, education and public-facing materials. Encourage businesses to consider the value-added by including ACP promotion with their clients.	
Standardize ACP terminology across the country	Education, Environmental restructuring
Encourage national harmonization of terminology. Use lay language. Avoid acronyms.	

## Discussion

Despite increasing international consensus on the benefits of ACP [15], low levels of public engagement in ACP remain a concern [15]. With aging populations giving rise to an increase in chronic disease, disability and dementia, earlier engagement in ACP is essential. In its landmark *Dying in America* report, the Institute of Medicine championed a “whole-community” approach to the promotion of ACP [16]. Similarly, Fried and Drickamer posit that garnering public support for and participation in ACP requires increasing public awareness of ACP through broad outreach [17]. Preparing community organizations with the tools they need to facilitate engagement in ACP in their communities was identified as a key goal by the Canadian ACP Task Group [18], and we see this as an integral step in promoting and sustaining public participation in ACP beyond the reach of our academic and healthcare organizations. We hypothesized that community groups (e.g. social, recreational, faith-based, disease support) would be particularly attractive targets for health promotion activities from sustainability, cost effectiveness and social diffusion perspectives. Based on the social nature of ACP and the universality of aging, illness and dying, it is well suited to group engagement.

The World Café method was a valuable forum for gathering input from community group members on how to increase public participation in ACP. To the best of our knowledge, this has not been reported previously, although other methods of engaging community groups in ACP have been described [19–24]. In general, we identified broad support for community groups to help promote ACP in Alberta. The main concerns of participants regarding sharing ACP with Albertans pertained to process issues, public awareness/knowledge of the topic, legal concerns and emotional concerns, among other issues. Many of these concerns could potentially be alleviated by widespread education efforts, provision of resources, and community group training and facilitation opportunities. This is supported by our finding that the majority of qualitative comments mapped to the Physical Opportunity and Psychological Capability domains, indicating a need for more/easier access to physical resources (e.g. Green Sleeves, personal directives, newsletter content, access to speakers and/or facilitators) and the knowledge/psychological skills to support ACP engagement. Some of these identified community needs are already being addressed and have evidence of their efficacy, for example group, public education sessions on ACP are offered by health services educators in Alberta and elsewhere [25–30]. Nevertheless, some issues would be harder to address at the community group level (e.g. healthcare provider time constraints), and would require multi-faceted interventions with multiple stakeholders and healthcare systems, beyond the provision of education and resources. Community organizations are positioned to raise awareness around the topic of ACP, and can encourage people to converse with their healthcare providers for ACP discussions. However, those healthcare providers and the systems of care need to be able to respond with skilled facilitative communication, including eliciting a person’s values and priorities, sharing prognosis, potential illness trajectories and outcomes, and having these conversations and plans accessible when they need to be reviewed at the point of care.

Participants were keen to suggest ways in which their community organizations could help share ACP with their members, which primarily involved education and training opportunities and provision of resources. These results are similar to those of Matthiesen et al., who found that community organizations were keen to engage with the issue of end of life care conversations, but needed accessible information and resources [19]. Outreach efforts to raise public awareness, and education for both the public and community group members, were also seen as priority areas for ensuring active community engagement work [19]. Many additional suggestions to improve community engagement in ACP put forth by our World Café participants are echoed in the work of other community engagement initiatives, such as simplifying language and forms [21, 24], normalizing conversations over the course of one’s life [20], and educating and financially compensating healthcare providers [20].

This World Café activity constitutes one part of a larger body of work by the ACP CRIO research program to explore Albertans’ perspectives and readiness for participation in ACP. The findings from this World Café are generally consistent with the results of our studies exploring ACP with patients, families and healthcare providers in several clinical settings and with the public in different sociocultural contexts. For example, interviews and focus groups with members of Calgary, Alberta’s South Asian communities revealed several common themes and suggestions, such as providing more educational opportunities in the community, making resources more widely available in community settings, using personal narratives and linking ACP with life events [31]. A synthesis of the findings from interviews with patients, family members and healthcare providers in renal/heart failure, cancer, and supportive living settings echo the recommendations to make use of personal experiences to increase personal relevance and understanding, to raise ACP awareness among the general public so that people are better prepared for ACP conversations, and to better prepare and support healthcare providers to engage in ACP with their patients. Finally, our recent survey of Alberta lawyers highlighted their concerns about lack of client preparedness for ACP and identified lawyers’ desire for more informational resources [32]. These consistent findings support a need for action in our communities to help normalize participation in ACP, as a first step toward ensuring people receive medically appropriate healthcare that is most concordant with their wishes and values.

Participant input across the World Cafés was used to generate eight recommendations for engaging Albertans in ACP. The recommendations generated vary in scope, cost and feasibility but a common theme is a need for cross-sector collaboration. Some recommendations are tangible and require minimal investment e.g. creation and dissemination of a series of “ready to use” newsletter pieces on the why, what and how of ACP for use by community groups. Using stories and personal reflection speaks to the content and method of ACP messaging. Other recommendations are more multi-jurisdictional e.g. standardizing terminology for legal documentation across Canada. These recommendations have been shared widely with a variety of stakeholders, along with which organizations are best placed to act on them. One way to move from these recommendations to *action* on public engagement in ACP could be the creation of an ACP working group for Alberta with representation from the multiple identified stakeholders. This could be similar to the Community-based Coalition Group model described recently by Waller et al. as part of their community action approach to promote adoption of ACP in the wider community [33]. With appropriate terms of reference and modest funding/resources this group could prioritize the recommendations, create action plans and initiate necessary changes and collaborations across sectors.

### Strengths and limitations

An inherent strength of the World Café method is its design to purposefully bring together a variety of stakeholders around a specific conversation [34]. In our experience, this activity brought together a diverse group of community members who would otherwise not typically interact with each other, to develop a community engagement strategy for ACP in Alberta.

An additional strength is our application of Michie et al.’s BCW [13] as a theoretical underpinning to identify candidate intervention strategies based on the World Café feedback. To the best of our knowledge, we are not aware of the BCW having been applied to a community engagement activity such as the World Café. As Michie and colleagues have described, many intervention strategies are designed without evidence of having assessed the nature of the behavior to be changed, the types of interventions that might match the behavioral target, the target population, and the context in which the intervention will be delivered [13]. As there is evidence that behavior change interventions informed by theory are more effective than those that are not [12], we posit that by applying this theoretical framework, the identified intervention functions are more likely to be effective in increasing public engagement in ACP.

This study has several limitations. First, as a qualitative inquiry, the findings are the specific views of café participants and cannot be generalized to any larger population. While participants were not expected to be ‘representative’ of their respective community group, we sought to ensure that a variety of perspectives were explored, and indeed we were pleased with the diversity of community organizations on behalf of which participants attended. We also note that many of the ideas generated by these participants are not unique to our locale or to Canada [35]. Second, those who self-selected to participate in the World Cafés may have had a greater interest in the topic or a vested interest in having their voices heard as compared to those who did not or could not attend. Third, the World Cafés were conducted in English such that participants needed to be English-speaking, and so the perspectives of other groups may be missing. However, the World Café method itself could be reproduced with other languages and populations, with resources available in several languages [7].

## Conclusions

The results of this activity suggest that community groups are well placed and keen to help promote ACP beyond the healthcare system. In keeping with the World Café philosophy, which emphasizes moving from talking to taking action, the results of the World Cafés have been used to generate several recommendations for engaging Albertans in ACP.

## Data Availability

The datasets used and/or analysed during the current study are available from the corresponding author on reasonable request.

## Abbreviations

ACP CRIO: Advance Care Planning Collaborative Research and Innovation Opportunities program
ACP: Advance care planning
BCW: Behavior Change Wheel
COM-B: Capability, Opportunity and Motivation behavior change system
GCD: Goals of Care Designation
TDF: Theoretical Domains Framework
